# Latinx attitudes, barriers, and experiences with genetic counseling and testing: A systematic review

**DOI:** 10.1002/jgc4.1632

**Published:** 2022-10-27

**Authors:** Heather A. Dron, Daiana Bucio, Jennifer L. Young, Holly K. Tabor, Mildred K. Cho

**Affiliations:** ^1^ Stanford Center for Biomedical Ethics (SCBE) Stanford University Stanford California USA; ^2^ University of Michigan Ann Arbor Michigan USA; ^3^ Providence St. Joseph Health Burbank California USA; ^4^ Center for Genetic Medicine Northwestern University Chicago Illinois USA; ^5^ Departments of Medicine, and by courtesy, Epidemiology Stanford University Stanford California USA; ^6^ Department of Pediatrics Stanford University Stanford California USA

**Keywords:** acculturation, barriers, communication, decision‐making, disparities, genetic counseling, genetic testing, Hispanic, Latinx, patient experience, systematic review, underrepresented populations

## Abstract

As genetics is increasingly used across clinical settings, there is a need to understand the impact and experiences of diverse patients. This review systematically examined research literature on Latinx experiences with genetic counseling and genetic testing (GC/GT) in the United States, synthesizing key themes and knowledge gaps pertaining to both patient experience and hypothetical scenarios. Findings were based on a systematic search, inclusion, and thematic analysis of 81 empirical peer‐reviewed articles published from January 1990 to July 2019 pertaining to Latinx populations and GC/GT. Studies most commonly addressed Latinas' perspectives on GC/GT in prenatal settings or for hereditary breast and ovarian cancer (HBOC). Costs, referrals, and communication were significant barriers to accessing genetic services for many Latinx patients, particularly those with low English proficiency (LEP). Studies highlighted difficulties accessing and communicating in healthcare settings, and how medical context and prior experience with healthcare workers and institutions influenced GC/GT decision‐making. Providers' implicit biases about Latinx patients negatively impacted their care and impeded communication. Despite low awareness of cancer GT, Latinx patients often reported interest in learning more about GC/GT or unmet needs for GT discussion and provider involvement. This systematic review identified areas where providers can take action to improve Latinx experiences with GC/GT. Clinicians should elicit and respond to patient preferences about shared decision‐making. For patients with low numeracy or LEP, providers should consider tailored educational and communication techniques. Most studies focused on HBOC and prenatal testing, and Latinx patients are heterogeneous, leaving many research questions about Latinx experience with GT/GC in other clinical areas.

**What is known about this topic**

Latinx patients may encounter additional barriers to genetic counseling and testing (GC/GT) and difficulties communicating across language and cultural differences, particularly with non‐directive approaches, highly technical language, or hypotheticals sometimes used in genetics. Fatalism and faith might influence receptivity to screen programs for cancer or birth defects. Latinx patients report competing obligations and effects on family members as particularly important factors in GT decision‐making, and despite generally low awareness, often see GT as an empowering tool for families.

**What this paper adds to the topic**

In the context where genetic testing is becoming a more routine part of medical care, a better understanding of Latinx patient experiences, barriers, and attitudes toward GC/GT may guide provider approaches and help ameliorate health disparities. Using expansive and systematic search criteria and a thorough review of full‐text English articles followed by qualitative thematic analysis, this manuscript provides an overarching synthesis of empirical research on Latinx patient perspectives, experiences, and barriers to GC/GT. In addition to highlighting patient‐level challenges such as difficulties communicating across language and culture or low awareness of GT and low health literacy or numeracy, this review addresses how medical context might support Latinx patients with risk‐appropriate GC/GT.

## 1 INTRODUCTION

Precision medicine interventions such as genetic counseling and genetic testing (GC/GT) aim to tailor medical management for patients based on genetic variation and other factors that affect patient health (e.g., environment and behavior). Despite GT's potential to decrease morbidity and mortality by identifying individuals at increased risk of disease and facilitating early, tailored, prevention and intervention, there are concerns that health disparities may be exacerbated by differential access to, or utilization of, new genetic applications (such as markers correlating with high disease susceptibility or better treatment approaches) (Sankar et al., 2004). Patients' experiences with genetic services affect their use, so understanding the experiences of diverse patient populations could guide prevention, provider approaches, and education strategies (Canedo et al., 2019).

The Latinx population is the largest ethnic minority in the United States (U.S.) with 18% of Americans self‐identifying as Hispanic in 2016 (Vespa et al., 2020). Definitions vary, but here Latinx is a gender‐neutral term used to define U.S. residents who self‐identified as having Latin American or Caribbean origins (sometimes called Latinos). Although Latinx are often portrayed as immigrants, only a third were foreign born in 2017 (Noe‐Bustamante, 2019). Some Latinx families predate U.S. western expansion and more recently, a declining proportion have nativity outside the U.S. (Vespa et al., 2020).

Epidemiologists have identified a Latinx health paradox, wherein for some health metrics they appear healthier and to have greater longevity than non‐Hispanic whites (NHW) (Ruiz et al., 2013). Despite debates about appropriate definition and use, acculturation, typically characterized by language preference and time of residence in the U.S., is used in health science research in an effort to understand these seemingly paradoxical differences in Latinx health outcomes (longer residence in the U.S. correlates with greater healthcare utilization but does not necessarily correlate with better health outcomes) (Hunt et al., 2004; Lara et al., 2005). If these outcomes reflect underlying genetic, socioeconomic, and cultural diversity, evidence suggests that, in the aggregate, this population has differential access to and use of genetic services, typically reported in cancer and reproductive healthcare (Avilés‐Santa et al., 2017; Cruz‐Correa et al., 2017; Lynce et al., 2016).

Like other marginalized groups, Latinx patients may be affected by discrimination, racial segregation, lack of insurance, substandard or unstable housing, dangerous work, or poverty (Berchick et al., 2018; Canedo et al., 2019). Older clinical summaries of GC for Latinx patients highlighted confusion with non‐directive approaches, challenges communicating across language and cultural differences, and mistrust born of persistent negative experiences, including racism, in healthcare settings (Penchaszadeh, 2001; Thorngren, 1990). Most GC/GT patient experience research has relied on populations of European descent, so less is known about the experience of racial and ethnic minorities (Canedo et al., 2019; Catz et al., 2005; Chavez‐Yenter et al., 2021).

Existing research indicates that cultural beliefs, such as faith or fatalism, might impede screening or familial communication about cancer or other intractable illnesses, but Latinx patients often had a high interest in GT and were motivated by the prospect of helping family members (Etchegary et al., 2013; Ricker et al., 2018; Southwick et al., 2020; Sussner et al., 2015). It can be difficult to assess whether there is an underlying Latinx cultural disinclination to prioritize predictive testing for birth defects or cancer in the face of competing life concerns and a sense of well‐being, or whether lower GT uptake, where it exists, reflects lower GT familiarity, poor communication and experiences in care settings, or lower referral, health insurance coverage, and access to healthcare (Rajpal et al., 2017; Southwick et al., 2020; Sussner et al., 2015). Researchers have highlighted communication challenges that low‐income monolingual Spanish speakers encounter in GC/GT, particularly with the interpretation of hypotheticals and technical language (Gutierrez et al., 2017; Joseph & Guerra, 2015; Kamara et al., 2018). When interviewed, immigrant Latina prenatal patients reported positive experiences of GC/GT, were encouraged to test by supportive medical contexts, and appreciated provider involvement to provide attentive supportive care (Browner et al., 1999; Garza et al., 2020; Molina et al., 2019; Trainor et al., 2020). Spanish‐speaking Latina breast cancer patients had “unmet need” for GT discussion (Jagsi et al., 2015). For English‐speaking Latinas and NHW with similar sociodemographic backgrounds and equivalent attitudes towards and receipt of information about BRCA 1/2 testing, lower perceived risk and lower GT awareness persisted among Latinas surveyed (Gammon et al., 2011). While there is extant Latinx‐specific patient experience research in reproductive and cancer care, the lack of Latinx perspectives on other types of GT, particularly those affecting men, requires an expansive approach including hypothetical studies and comparative research on barriers to and attitudes toward GC/GT in diverse clinical areas. Responding to knowledge gaps, this systematic review uses existing empirical literature to characterize Latinx GC/GT attitudes, barriers, and experiences amidst an expansion of GT in diverse clinical contexts.

## 2 METHODS

Following the methodology of a prior review of GC/GT with Asian Americans (Young et al., 2021), we comprehensively searched research literature on Latinx patient experience and summarized it thematically. Abstract and title word searches and MESH terms were used to query literature in five databases: PubMed, Embase (Elsevier), CINAHL (EBSCO), PsychInfo (ProQuest), and Social Services Abstracts (EBSCO). Peer‐reviewed articles were included if they had at least one of several terms corresponding to Hispanic or Latinx populations (e.g., Latinos/as) *and* one associated with GC/GT.

After identifying articles, the team reviewed titles and abstracts based on the following inclusion criteria: peer‐reviewed empirical publications in English, published from January 1990 to July 2019, that addressed adults or children receiving GC/GT (including experiences with GC/GT, observations of pediatric genetic services, perceptions of GC/GT, prenatal testing, or provider's observations of patients receiving GC/GT) *and* studied Hispanic or Latinx population (>9% of study population and a specific finding). For example, studies that interviewed patients about amniocentesis or breast cancer screening were included, as were surveys of Latinx attitudes about genetics. Studies focused on clinical outcomes or the geography of genetic variation were not included.

Articles with the following characteristics were excluded: not in English, no peer review, no original data (e.g., reviews), unavailable full text (e.g., conference abstracts), or inappropriate study design, patient population, or outcome (e.g., operations research or clinical summaries). Using Covidence software, the first author (H.A.D.) examined titles and abstracts for relevance. Further review and selection of articles were conducted by a two‐person team (D.B. and H.A.D.) that reviewed the full texts and made inclusion decisions. Differences, involving roughly 20% of studies, were resolved through discussion with another researcher (H.K.T., J.L.Y., or M.K.C.). Data extraction was performed by the first author (H.A.D.) using Excel software, collecting information on geographical and clinical settings; sample characteristics (e.g., patient or provider and pregnancy status); gender and ethnicity labels; study aim, design, and sampling approach; methodology and measurement tools; and a summary of findings. Data and methods reporting was based on PRISMA standards, and analysis was informed by a grounded theory and JBI convergent integral mixed methods approach to integrate findings and identify qualitative themes despite methodological differences (Charmaz, 2014; Page et al., 2021; Young et al., 2021). In practice, this entailed detailing and coding key findings from each study, then reordering and ranking them by frequency to identify common patterns across studies. Themes were identified irrespective of the clinical context to capture the breadth of research across clinical areas, although cancer and prenatal GC/GT studies were most frequent. This article synthesizes results on U.S.‐based populations using the term Latinx.

## 3 RESULTS

### 3.1 Characteristics of studies and study populations

**FIGURE 1:**
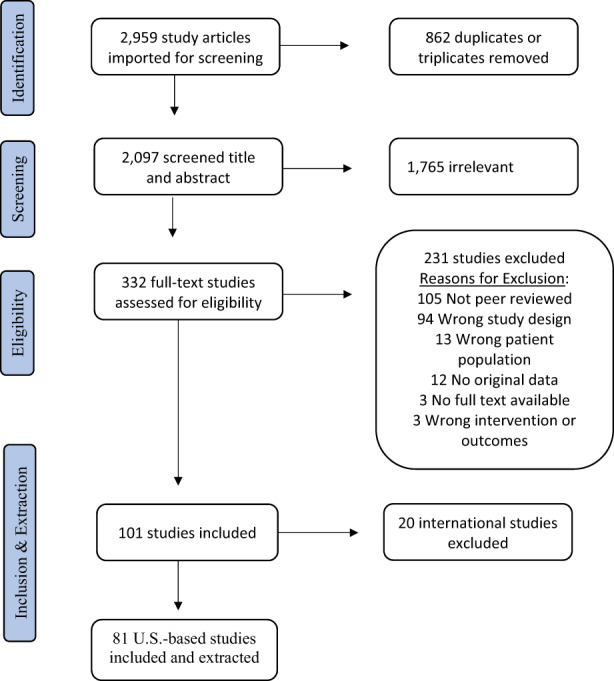
Search and selection of studies based on PRISMA criteria

In total, 81 studies on Latinx patient experience with genetic services in the U.S. were included in this analysis, of 332 eligible from 2097 unique articles (Figure 1). These 81 included studies were selected after a full‐text review of 194 empirical peer‐reviewed U.S.‐based manuscripts in English (see Appendix A). Of included articles, 44% (36) were quantitative, 38% (30) were qualitative, and 17% (14) deployed mixed‐method study designs. Over half (43) queried the experiences of patients or at‐risk family members, while a third (27) asked Latinx community members about hypothetical clinical scenarios or perspectives on GT. The remaining 12 studies addressed parents' perspectives pertaining to pediatric patients (10%) or providers' experience with Latinx patients (5%).

Three‐fourths of the studies (61) had participant populations that were > 60% female, while 51% (41) of studies restricted recruitment to women and 27% (22) sampled only pregnant women. The terms Hispanic and Latinx/Latinos were typically used interchangeably; less than half (38) provided more detailed information on ethnicity, and only 15% (12) restricted their analysis to more specific ethnicities defined by geographical or national origins, primarily Mexico or Puerto Rico (Browner et al., 1999; Vadaparampil et al., 2011; Withers et al., 2019). Three studies defined groups by preferred language, comparing Spanish speakers to English speakers (Catz et al., 2005; Hawk et al., 2011; Raymond, 2014). Over half of the studies (45) also collected data on participants from a racial group and 31% (25) reported comparisons between people of Hispanic or Latinx ethnicity and a racial group, often NHW, using a range of terms. For example, studies compared Black and Hispanic populations, although some participants may identify as both.

**FIGURE 2:**
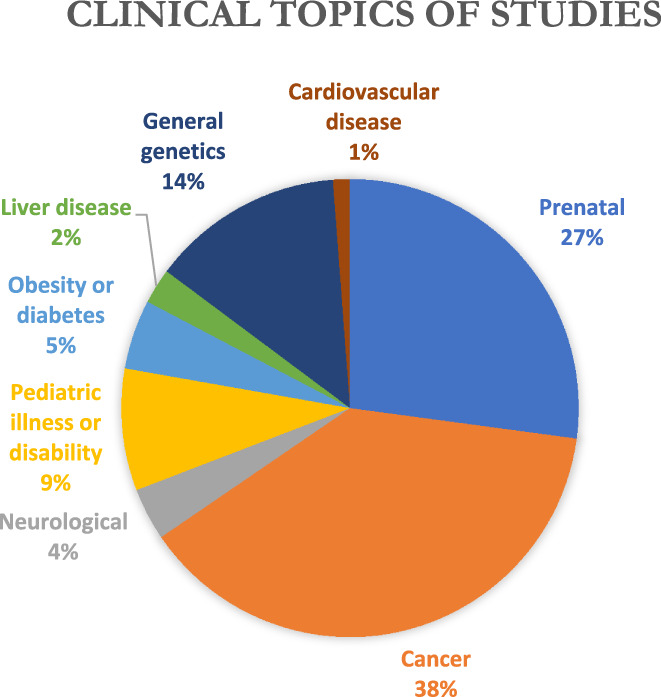
Clinical topics of studies

Of the clinical areas specifically addressed (see Figure 2), 38% (31) of the articles were on cancer and 27% (22) on prenatal GC/GT. Other conditions were addressed in a small fraction of articles: pediatric illness or disability (9%), diabetes or obesity (5%), neurological disorders (2%), liver disease (2%), cardiovascular disease (1%), and non‐specific GT (14%).

### 3.2 Qualitative summary of key findings

Studies addressed numerous topics related to Latinx patient experience, clustering around five common themes: (1) knowledge, awareness, information sources, and interest in GC/GT; (2) language, patient–provider, or intrafamilial communication; (3) acculturation and the impact of Latinx culture on GC/GT; (4) barriers and facilitators to GC/GT; and (5) provider recommendations. Several subthemes further organize the findings to illustrate high interest despite generally low Latinx GT awareness, key knowledge sources, challenges of communication and referral across language and culture, and the influence of fatalism, family, and faith.

## 4 KNOWLEDGE, AWARENESS, INFORMATION SOURCES, AND INTEREST

### 4.1 Low awareness but high interest

Across studies, Latinx participants reported low knowledge of GC/GT. Nationally representative telephone surveys documented that awareness of GT among Latinx respondents in the early 2000s was less than half that of NHW (Heck et al., 2008; Ortiz et al., 2011; Pagan et al., 2009; Vadaparampil et al., 2006; Wideroff et al., 2003). Measures of greater acculturation and higher income correlated with greater cancer GT familiarity, and preference for Spanish was a strong predictor of lower awareness (Heck et al., 2008; Sussner et al., 2009; Vadaparampil et al., 2006). Those with a personal or family history of cancer reported little knowledge of genetics and GT (Cragun et al., 2017, 2019; Heck et al., 2008; Vadaparampil et al., 2010). Even those with prior cancer GC/GT reported little education about GT and had trouble articulating their cancer risk (Rajpal et al., 2017; Ramirez et al., 2006, 2015).

Most studies reported high interest in undergoing GT among participants (Case et al., 2007; Catz et al., 2005; Chalela et al., 2012; Glenn et al., 2012; Kaphingst et al., 2015; Kinney et al., 2010; Singer et al., 2004). Many participants were Latinas with a personal or family history suggestive of hereditary breast or ovarian cancer (HBOC), who expressed more interest in GT than participants of other ethnicities (Jagsi et al., 2015). Latinx participants at risk of HBOC viewed the benefits of GT to be greater than the risks or limitations; in other words, they viewed GT positively, irrespective of prior knowledge (Ramirez et al., 2006).

### 4.2 Purported low knowledge

Studies of Latinx patients and GC/GT emphasized patient‐level limitations, including low genetics knowledge and numeracy, even after GC (Eichmeyer et al., 2005; Mittman et al., 1998; Saucier et al., 2005; Steinberg et al., 2007). Some researchers identified patients' preferred information sources or evaluated educational and counseling approaches that might ameliorate this apparent deficit (Hawk et al., 2011; Kinney et al., 2010; Sussner et al., 2010, 2013). Another study attributed racial or ethnic differences in genetic term familiarity to lower health literacy, which also correlated with Latinx ethnicity (Williams et al., 2018).

### 4.3 Education, media, and preferred information sources

Instead of online or kiosk‐based approaches, Latinx patient populations preferred printed materials about GC/GT in non‐technical language or visual depictions (Catz et al., 2005; Eichmeyer et al., 2005; Griffiths & Kuppermann, 2008; Vicuña et al., 2018). Latinx patients were less likely to consult the internet or libraries when compared to other patient populations and some reported obtaining information through discussion with family and friends (Browner & Preloran, 2000; Catz et al., 2005; Vicuña et al., 2018). In cancer care and prenatal settings, participants reported that they wanted materials with disease‐specific information that avoided jargon or technical language, as providers sometimes emphasized information not relevant to patient decision‐making (Hunt et al., 2005; Kinney et al., 2010). Spanish speakers, in particular, desired more fundamental information about the testing process (Floyd et al., 2016; Hawk et al., 2011).

## 5 LANGUAGE, PATIENT–PROVIDER, AND INTRAFAMILIAL COMMUNICATION

Researchers reported that Latinx patients were often deferential to medical authority or recommendations, and healthcare providers were an influential and trusted source of medical advice about GC/GT (Chalela et al., 2012; Hamilton et al., 2016; Hurtado‐de‐Mendoza et al., 2018; Ramirez et al., 2015). Several studies indicated that biases about Latinx patients among providers affected patient–provider communication. For example, if providers assumed that Latinx patients had deeply rooted and non‐negotiable cultural beliefs, particularly about abortion, in‐depth counseling about medical decisions seemed unnecessary and might be omitted (Browner et al., 1999). Patients sometimes assumed that providers would provide directive advice (Mittman et al., 1998), and non‐directive counseling was confusing or misinterpreted (Browner & Preloran, 2000; Hurtado‐de‐Mendoza et al., 2018; Joseph & Guerra, 2015). Studies of prenatal experiences indicated that patients interpreted repetition of questions about a medical intervention (primarily amniocentesis) as subtle pressure to have the intervention; or alternatively, perceived lack of a clear medical recommendation or prescriptive advice to mean that no action was required (Browner et al., 1999, 2003). Spanish‐speaking women and those with lower health literacy more frequently reported a mismatch between their stated preferences about prenatal GT decision‐making and their provider's level of involvement, in part because many preferred more clinician involvement in decision‐making (Molina et al., 2019).

Studies on cancer predisposition testing reported that Latinx patients, particularly monolingual Spanish speakers, were less likely than NHW to discuss testing with or communicate genetics‐related information (family history and results) to providers (Hurtado‐de‐Mendoza et al., 2018; Jagsi et al., 2015; Ricker et al., 2018). For instance, Latinx patients were less likely to report having told a provider about likely pathogenic cancer genetic variants when compared to NHW (29% vs. 68%) (Ricker et al., 2018). Translation of technical terms could be lengthy and result in misunderstandings, omissions, and misinterpretations (Gutierrez et al., 2017; Rodríguez et al., 2017). Providers reported difficulty obtaining accurate family medical histories for Latinx patients due to vague responses, abbreviated sessions in non‐concordant languages, lack of knowledge of family health history, or dispersed relatives without access to comprehensive care (Hamilton et al., 2016; Hurtado‐de‐Mendoza et al., 2018; Joseph & Guerra, 2015).

There is somewhat contradictory evidence about Latinx intrafamilial communication about family disease history or GT results. When asked who has a right or responsibility to disclose genetic information, in a few studies, Latinx respondents reported more support for direct physician disclosure of genetic information to family members than NHW (Kinney et al., 2010; Singer et al., 2004). Latinx opinions on intrafamilial sharing of GT results were variable (Frazier et al., 2006; Kaphingst et al., 2015), showing similarly reported sharing of cancer GT results by Latinx and NHW respondents, but not for hemochromatosis, a hereditary iron absorption disorder which causes serious health effects for a minority of affected individuals (Ricker et al., 2018; Tucker et al., 2006). Even for cancer, there were conflicting tendencies: a sense of obligation to use cancer GT information for family benefit (Etchegary et al., 2013; Fehniger et al., 2013; Hamilton et al., 2016; MacDonald et al., 2008; Ricker et al., 2018; Tucker et al., 2006), even as some Latinx patients wanted to hide stigmatized disease diagnoses (Kinney et al., 2010).

## 6 ACCULTURATION AND IMPACT OF LATINX CULTURE ON GC/GT

### 6.1 Acculturation

Some studies set out to understand how cultural beliefs affected patient decision‐making, awareness, attitudes, or utilization of GC/GT. Greater acculturation was linked to greater awareness and perceived benefits of GT for cancer risk (and fewer perceived barriers), and was not correlated with perceived disadvantages of GT (Heck et al., 2008; Pagan et al., 2009; Sussner et al., 2009; Vadaparampil et al., 2006).

Latinas declined prenatal testing more frequently than patients of other ethnicities during the expansion of testing in California in the 1990s, which made some providers assume they were religious, fearful of medical procedures, fatalistic, superstitious, and heavily influenced by family members and male partners (Hunt et al., 2005; Hunt & de Voogd, 2005; Rapp, 1993). Spanish‐speaking women with lower acculturation, or those born in Mexico, were initially more likely to decline prenatal testing (Browner et al., 1999; Press & Browner, 1998). Other studies showed that perceptions of benefits and risks, patient–provider communication, the healthcare context, and relationships with healthcare workers influenced medical decision‐making as much as cultural beliefs, with patients more willing to undergo interventions in settings where they felt comfortable or in an effort to maintain rapport with providers (Browner et al., 1999; Mittman et al., 1998; Press & Browner, 1998).

### 6.2 Loyalty to family

Several studies identified the importance of family support for Latinx patients facing illness (Sheets et al., 2012; Sussner et al., 2015). Yet, when providers in prenatal settings directed medical information toward family members or assumed that Latinx patients are influenced by family—such as when a family member interpreted or a patient was told to consult their family or partner before making a decision—it sometimes undermined patient autonomy by omitting clear communication and agreed on a course of action between patient and provider or delaying a decision pending input (Browner et al., 1999; Hunt & de Voogd, 2005, 2007). In fact, many Latinas encouraged their partner's involvement in prenatal GT decisions to reinforce shared parenting, even as they referenced bodily autonomy and characterized their discussions with male partners or family as consultations, not requests for permission (Hunt & de Voogd, 2005; Susan Markens et al., 2003).

Nevertheless, Latinx patients were more motivated to test for perceived familial benefit when compared to NHW and African Americans (AA) (Singer et al., 2004). Numerous studies underscored respect for family ties, as one perceived benefit of GT was to use results (usually about cancer risk or birth defects) to inform their children, for family planning, or to help other family members (Carmichael et al., 2017; Chalela et al., 2012; Cragun et al., 2019; Cuccaro et al., 2014; Glenn et al., 2012; Heck et al., 2008; Hurtado‐de‐Mendoza et al., 2018; Palmer et al., 2008, 2009; Rajpal et al., 2017; Ramirez et al., 2006; Sussner et al., 2010; Vadaparampil et al., 2011; Withers et al., 2019). For instance, helping family and family planning were more important for Latinx vs. NHW parents when considering GT for deaf infants (Palmer et al., 2008). Latinas expressed more worry about the impact of high genetic risk status (for diabetes and cancer) on their family members than NHW, yet also saw informing descendants (particularly daughters) about cancer risk as a benefit of HBOC GT (Bennett Johnson et al., 2004; Jagsi et al., 2015; Rajpal et al., 2017; Ramirez et al., 2006). The desire to help family members may be accompanied by a sense of guilt when communicating positive results, and some Latinx research participants expressed dismay about extensive predictive GT for children (Hickey et al., 2014; Streicher et al., 2011).

### 6.3 Religion, fatalism, or disease stigma

Culture‐specific stigma, shame, and secrecy about intractable illnesses, particularly cancer, can prevent sharing disease‐related information in Latinx families (Joseph & Guerra, 2015). Studies of cancer prevention reported that Latinx populations avoided screening because of *fatalismo* (fatalism), a sense that the disease trajectory was determined by fate, and that little could be done (Chalela et al., 2012; Kinney et al., 2010; Rajpal et al., 2017; Sussner et al., 2013, 2015). Nevertheless, cultural beliefs that impede cancer screening may largely affect older patients (Rajpal et al., 2017; Ramirez et al., 2006; Sussner et al., 2015), and Latinas engaged in care reported low fatalism, such as those at risk of HBOC surveyed before or after appointments (Lagos et al., 2008; Macdonald et al., 2012). In the context of prenatal screening, Latinas mentioned divine will, the evil eye (*mal de ojo*), or curses as causes of disability, implying stigma and inability to intercede (Griffiths & Kuppermann, 2008; Kinney et al., 2010; Mittman et al., 1998; Sheets et al., 2012).

Many Latinx patients reported deriving support from religious communities or faith in God, often mentioned in prenatal or pediatric settings (Browner et al., 1999; Seth et al., 2011; Sheets et al., 2012; Steinberg et al., 2007; Sussner et al., 2015). Religious background and belief in church teaching on reproduction have been significantly associated with prenatal testing refusal (Press & Browner, 1998), and were sometimes seen as inconsistent with GC/GT (Sussner et al., 2010; S. Thompson et al., 2015). Yet, many women underwent amniocentesis irrespective of their religious beliefs or opposition to pregnancy termination (Hunt & de Voogd, 2005). Latinas' references to God during prenatal screening carried different meanings, sometimes indicating that birth defects were subject to divine will, and thus outside of their power to control, and sometimes underscoring that despite faith they did not put all their trust in God (Browner & Preloran, 2000; Griffiths & Kuppermann, 2008). Religion might be a source of comfort but was not determinative of GT decision‐making (Browner et al., 1999; Saucier et al., 2005; Seth et al., 2011).

## 7 BARRIERS AND FACILITATORS TO GC/GT

### 7.1 Barriers

Even as they reported high interest in GT, Latinx patients experienced barriers from structures beyond their locus of control. The most commonly cited barriers to GC/GT, not unique to Latinx patients, were financial concerns such as cost and insurance coverage for GT/GC (Carmichael et al., 2017; Chalela et al., 2012; Cragun et al., 2019; Gammon et al., 2011; Glenn et al., 2012; Hamilton et al., 2016; Hurtado‐de‐Mendoza et al., 2018; Kinney et al., 2010; Rajpal et al., 2017; Sussner et al., 2015; Vadaparampil et al., 2010, 2011; Wagner et al., 2018). Latinx patients had more expectations of personalized GC conversations about insurance than NHW (Wagner et al., 2018). Again, low awareness of GT was a barrier, which correlated with lower acculturation (Cragun et al., 2017; Heck et al., 2008; Hurtado‐de‐Mendoza et al., 2018; Rajpal et al., 2017; Sussner et al., 2009, 2015).

Another reported barrier, albeit sometimes motivating, was emotional distress associated with decision‐making or the possibility of getting a positive GT result (Bennett Johnson et al., 2004; Browner et al., 1999; Catz et al., 2005; Glenn et al., 2012; Hamilton et al., 2016; Hickey et al., 2014; Jagsi et al., 2015; Kinney et al., 2010; Rajpal et al., 2017; Ramirez et al., 2006; Sussner et al., 2010, 2015; Vadaparampil et al., 2010; Witt et al., 1996). Latinx participants, like patients of other ethnicities, described distress, anxiety, worry, fear, helplessness, and shame resulting from making a decision about testing or finding out disease risk information for themselves, their fetus, or their family (Bennett Johnson et al., 2004; Browner et al., 1999; Catz et al., 2005; Glenn et al., 2012; Hamilton et al., 2016; Hickey et al., 2014; Jagsi et al., 2015; Kinney et al., 2010; Macdonald et al., 2012; Rajpal et al., 2017; Ramirez et al., 2006; Sheets et al., 2012; Sussner et al., 2010, 2015; Vadaparampil et al., 2010; Witt et al., 1996). Participants cited concerns about invasive medical procedures or disinclination to prioritize personal health over family needs (Browner & Preloran, 2000; Glenn et al., 2012; Sussner et al., 2013).

In cancer care, lack of access to specialists, low rates of physician GT discussion, and low referrals were barriers to GC/GT (Cragun et al., 2017, 2019; Hurtado‐de‐Mendoza et al., 2018; Jagsi et al., 2015; Vadaparampil et al., 2010). This was compounded by the challenges of patient–provider communication across language differences and difficulties documenting familial disease history (Cragun et al., 2017; Hurtado‐de‐Mendoza et al., 2018; Pasick et al., 2016; Ramirez et al., 2015; Ricker et al., 2018; Sussner et al., 2013, 2015; Vadaparampil et al., 2010). Latinx breast cancer survivors were less likely to discuss genetic testing with a provider and receive a GT recommendation or referral than NHW (Cragun et al., 2017, 2019). Non‐genetics providers were unfamiliar with reimbursement for GC/GT, likely discouraging the referral of low‐income patients (Hurtado‐de‐Mendoza et al., 2018).

Decision‐making about GC/GT was influenced by attitudes about medicine and prior relationships with healthcare staff (Browner et al., 1999; Hamilton et al., 2016; Kukafka et al., 2015; S. Markens et al., 2010; Rajpal et al., 2017; Rapp, 1993; Sussner et al., 2015; Sussner et al., 2013; Sussner et al., 2009; Suther & Kiros, 2009; Thompson et al., 2003). Mistrust of providers was characterized by: (1) complaints that providers did not spend enough time, lacked empathy, or that they performed extraneous procedures to make money (Rajpal et al., 2017); (2) negative experiences with healthcare (long waits, impatient healthcare workers, and feeling blamed for inadequate care) (Steinberg et al., 2007; Sussner et al., 2015); (3) fears of healthcare rationing (Kukafka et al., 2015); or even (4) illicit and unconsented activities, like stealing or selling blood (Hamilton et al., 2016). Such perceptions discouraged risk‐appropriate GC/GT. Latinas were influenced to accept GT by supportive and respectful medical contexts or to maintain good relationships with healthcare staff (Browner et al., 1999; Rapp, 1993).

Some Latinx respondents were concerned about negative consequences, misuse, risks, or limitations of GT, which were influenced by characteristics of the population sampled, including age, religiosity, gender, and socioeconomic status (Catz et al., 2005; Hall et al., 2005; Hamilton et al., 2016; Palmer et al., 2008; Ramirez et al., 2015; Singer et al., 2004; Sussner et al., 2009; Suther & Kiros, 2009; Thompson et al., 2003). Some studies showed that Latinx populations were more likely to express concerns about unfair treatment or insurance discrimination (Glenn et al., 2012; Hall et al., 2005; Hamilton et al., 2016; Sussner et al., 2013, 2015), and were marginally more concerned about GT misuse and more doubtful that physicians would maintain confidentiality when compared to NHW (based on a survey of agreement that “information from GT is likely to be misused”) (Rajpal et al., 2017; Singer et al., 2004; Suther & Kiros, 2009). In contrast, other studies found no differences between concerns about discrimination among Latinx and NHW respondents or that greater Latinx insurance‐discrimination‐related concerns lost significance in regression models that adjusted for income and education, suggesting socioeconomic confounders (Carmichael et al., 2017; Hall et al., 2005). One study found that Latina respondents reported no concerns about HBOC GT risks while other groups reported four or more. Authors hypothesized that this difference may be influenced by *simpatia*, or a desire to have respectful and positive relationships with researchers and healthcare providers (Ramirez et al., 2015).

Lack of perceived risk or the perception that a sense of well‐being made GT unnecessary was cited as a barrier (Sussner et al., 2013; Thompson et al., 2003; Witt et al., 1996). Latinx community members reported that they did not need GT because they felt healthy more frequently than NHW and AA (Thompson et al., 2003). Many studies mentioned “corporeal concerns” and fear of unpleasant, invasive, dangerous, or painful interventions or testing processes (Carmichael et al., 2017; Thompson et al., 2015; Vadaparampil et al., 2011).

Several logistical and practical challenges were mentioned. Latinx community members, particularly those with LEP, faced significant difficulties accessing care and communicating in healthcare settings (Hamilton et al., 2016; Hurtado‐de‐Mendoza et al., 2018; Rajpal et al., 2017). At‐risk Latinas, particularly in urban settings, highlighted competing demands on their time such as caregiving (Glenn et al., 2012; Rajpal et al., 2017; Sussner et al., 2013).

As noted before, fatalism, shame, or secrecy associated with a disease diagnosis could be a barrier (Hurtado‐de‐Mendoza et al., 2018; Kinney et al., 2010) as Latinas sometimes reported more fatalistic perspectives about cancer than other groups (Hurtado‐de‐Mendoza et al., 2018; Rajpal et al., 2017), or anticipated feeling ashamed of positive GT results (Thompson et al., 2003).

### 7.2 Facilitators or motivators to GT

Studies underscored reasons, like other patient populations, that Latinx patients reportedly would undergo GT: improved prevention and early detection, to inform medical management, to inform children and other family members, to know recurrence risks or guide family planning, because a physician recommended it, and to seek reassurance (typically, that their fetus was healthy). The prospect of disease prevention or other health‐related utility, like treatment decision‐making, was salient (Carmichael et al., 2017; Catz et al., 2005; Glenn et al., 2012; Hurtado‐de‐Mendoza et al., 2018; Palmer et al., 2008, 2009; Steinberg et al., 2007; Vicuña et al., 2018; Witt et al., 1996). Depending on the condition, participants, especially older adults, reported motivation for healthier behaviors—such as for type 2 diabetes mellitus or obesity (Carmichael et al., 2017; Segal et al., 2007)—or, in the case of autism or autosomal‐dominant Alzheimer's disease, were more focused on preparation (Chen et al., 2015; Hamilton et al., 2016; Ramirez et al., 2006; Vicuña et al., 2018; Withers et al., 2019).

Reassurance or reduced worry was discussed, typically referring to the health of the fetus in prenatal and carrier screening—although identifying lower disease risk was an underrecognized benefit of GT for some adult conditions (Griffiths & Kuppermann, 2008; Hunt et al., 2005; Sussner et al., 2010, 2013). When compared to NHW, Latinx patients reported low cost, benefiting children and family, and physician discussion and recommendation as particularly important (Cragun et al., 2019; Jagsi et al., 2015; Vadaparampil et al., 2010). For this population, health insurance coverage and physician recommendation or referral remained important facilitators, augmented by co‐location of genetic services in a patient's healthcare setting (Cragun et al., 2019; Hurtado‐de‐Mendoza et al., 2018; Sussner et al., 2013; Vadaparampil et al., 2011).

## 8 RECOMMENDATIONS FOR PROVIDERS

Some of the literature gave recommendations for genetics providers (see summary in Table 1). Providers were often trusted authoritative figures for Latinx patients and non‐directive approaches could be confusing for those expecting a more prescriptive approach to medical decisions (Browner et al., 2003; Browner & Preloran, 1999; Chalela et al., 2012; Hurtado‐de‐Mendoza et al., 2018; Mittman et al., 1998). Some studies suggested Latinx‐specific approaches to patient–provider communication, such as acknowledging competing demands, emphasizing the interconnectedness of family and individual well‐being, and more provider involvement in prenatal decision‐making (Joseph & Guerra, 2015; Molina et al., 2019; Sussner et al., 2013). It may be difficult for providers to guide patients regarding a testing choice as steeped in personal values and uncertainty as one that may inform pregnant women about fetal disabilities, influencing pregnancy termination or parenting a disabled child, nevertheless, that was sometimes desired (Molina et al., 2019). Researchers recommended eliciting patient preferences about shared decision‐making, explicitly stating all choices, using open‐ended questions, and developing tailored educational programs or counseling tools aimed at those with LEP or low numeracy (Joseph & Guerra, 2015; Kamara et al., 2018; Molina et al., 2019; Rajpal et al., 2017; Wideroff et al., 2003).

**TABLE 1:** Key findings and recommendations

**Theme**	**Subthemes**	**Key Findings**	**Recommendations**
Knowledge	Low GT awareness Low numeracy Mismatch between technical information and patient psychosocial or informational needs	Preference for printed materials with visual aids or simplified language over internet sources. Provision of technical information may not be optimized to meet patient concerns.	Provide visuals or printed take‐home materials. Those with low numeracy or health literacy may appreciate more provider involvement. Focus on disease‐specific information over genetic science.
Communication	Language/Interpretation Expectation of directiveness Family involvement in interpretation affects medical decision‐making Providers reported incomplete family histories, particularly for immigrants or those with low English proficiency	Latinx patients may misinterpret non‐directive approaches. Some Latinx patients preferred more provider involvement and appreciated attentive care. Misunderstanding of key information was common, such as over‐ or underestimates of risk or conflation of GT with other types of routine medical testing. Latinx breast cancer survivors reported unmet needs for GT discussion. Spanish‐speaking Latinx patients had challenges communicating in healthcare settings, getting GT/GC referrals, and receiving preference‐concordant care. Providers reported difficulties eliciting family histories from Latinx patients. Untrained interpreters or family members may mistranslate technical terms.	Solicit patient preferences about shared decision‐making; non‐directive approaches may be confusing. Solicit specific psychosocial and informational needs. Avoid hypotheticals. Use teach‐back and jargon‐free language with LEP patients. Proactive trained interpreters aided informed consent, bridged information differences, and maintained patient engagement. When a family member interprets, ensure the patient receives complete disclosure and contributes to decision‐making.
Culture and acculturation	Variable acculturation *Fatalismo* (fatalism) or disease stigma *Familismo* (loyalty to family) Faith	Higher acculturation was associated with greater awareness of GT, preventative health behaviors, and access to healthcare (but not necessarily better health outcomes). Spanish‐speaking Latinas sometimes reported more worry about the implications of GT for children and other family members than NHW or English‐speaking Latinas. Family members and religion were sources of support that influenced but did not play a determinative role in decision‐making about GT. Disease stigma may discourage preventative behaviors, intrafamilial communication, or reporting complete family histories.	Latinx patients may be motivated by helping family. Providers should consider emphasizing the interconnectedness of personal and family health and well‐being. Explicitly state all options, including pregnancy termination.
Barriers /Facilitators	Cost of insurance coverage Low discussion and referral Difficulties reporting complete family histories Concerns about psychosocial effects of GT or decision‐making Negative prior experience with healthcare workers or institutions Perceived limitations of genetic testing Feeling healthy and fearing the procedure Competing demands, such as caregiving Facilitators include low cost, provider recommendation, and the prospect of aiding family and community. Physician recommendation and peer navigators as facilitators	Patients and non‐genetic specialists had uncertainty about GT/GC insurance coverage, which may affect referrals. Latinx patients expected more conversations about GC coverage than NHW. Latinx patients may think GC/GT is unnecessary if they are feeling well, particularly if a procedure is invasive and the perceived benefits of GT are low. Latinx patients sometimes exhibited skepticism, concerns about unfair treatment, or reported prior negative experiences with healthcare. Anticipated emotional responses to decision‐making or results could act as both a barrier and a facilitator to GT. Provider discussion and referral to GT is an important facilitator.	Discuss GC/GT options and insurance coverage with Latinx patients and refer them to risk‐appropriate GC/GT. Healthcare context and positive engagement with staff may facilitate uptake of GT. Consider a peer navigation program.

Researchers gave interventions tailored to socioeconomically or linguistically isolated groups (Wideroff et al., 2003), such as communication strategies for LEP patients that included: avoiding hypotheticals, simplified language, teaching back strategies, and omitting unnecessary technical information to respond to the informational and psychosocial needs of individual patients (Kamara et al., 2018; Sheets et al., 2012). Interpreters could act as a cultural or informational bridge (Kamara et al., 2018; Preloran et al., 2005; Raymond, 2014). Studies documented low numeracy and a tendency to misunderstand or overestimate risk (of birth defects, cancer mortality, adverse effects of amniocentesis, and pathogenic genetic variants) among Latinx populations, and a preference for visual references and take‐home printed materials in simplified language (Catz et al., 2005; Eichmeyer et al., 2005; Griffiths & Kuppermann, 2008; S. Thompson et al., 2015). A provider referral did not always mean patients followed through with GC/GT for HBOC, and Latinx patients cited competing logistical and familial demands. Thus, improved follow‐up, possibly with peer navigators emphasizing interconnections between family and personal well‐being, was recommended (Sussner et al., 2013, 2015).

## 9 DISCUSSION

Research synthesized in this review identified low awareness of GT, poor patient–provider communication, low referral for GC/GT, and concerns about potential harms among Latinx populations. At the same time, Latinx participants reported positive perceptions of and high interest in GT, and expectation of prescriptive medical advice. Despite the high interest, barriers reported included: concerns about cost or coverage; anticipated emotional response to testing results or decision‐making; lack of physician discussion or GT referral; prior negative experiences with healthcare; perceptions of limitations or disadvantages of GT; feeling healthy and fearing the procedure; practical and logistical challenges to getting care; and disease stigma or fatalism. Latinx respondents typically reported more concerns about discrimination, less communication with providers, and more worry about family members than NHW populations.

This review largely supports findings from prior research on Latinx patient experience with GC/GT. Challenges identified by prior summaries of GC for Latinx patients endure, including language gaps, misunderstandings, cultural differences, concerns about cost, institutional insensitivities, and providers' stereotypes, that impact Latinx patient experience of GC (Penchaszadeh, 2001; Thorngren, 1990). This review concurs with commonly cited barriers to GC among other racial and ethnic minorities, emphasizing logistics and effects on family members as of particular concern for Latinx patients (Cragun et al., 2017, 2019; Southwick et al., 2020). Findings from cancer care research highlighted the low use of genetic services among people of color, identifying low awareness, cancer stigma, and difficulties with effective cross‐cultural communication and referral as barriers (Hann et al., 2017; Lynce et al., 2016). From prenatal research, there is evidence that positive views expressed by Latinas about GT and interest in receiving GT does not translate into higher rates of amniocentesis among Latinx patients when compared to other groups (Case et al., 2007; Kuppermann et al., 2006). Research in which Latinas express positive uncritical perspectives toward GC/GT may be influenced by *sympatía*, or a desire to maintain positive relationships and build rapport with researchers or medical staff, which could also play a role in GT acceptance (Ramirez et al., 2015). Consistent with our findings in Latinx populations, recent studies that examined racial and ethnic differences in attitudes and knowledge about GT underscored low background knowledge among non‐white participants and Latinx interest in using GT for family benefit but they do not elaborate on the role of medical context (Canedo et al., 2019; Chavez‐Yenter et al., 2021; Langford et al., 2012).

In contrast to studies focused on patient challenges, such as low Latinx health literacy or numeracy, this review highlights the importance of the provider approach and medical context. Successful medical care involves a complex mix of structural, provider, patient, and institutional factors; however, studies focused on patient challenges sometimes do not convey how genetics provider assumptions or medical context affect Latinx patients' decision‐making about GC/GT. Providers and researchers sometimes assume that Latinx patients have low prior knowledge of genetics, mistrustful attitudes, and religious and cultural beliefs that impede GT acceptance, or that Latinx patients have low susceptibility to genetic disease. These perceptions coexist uneasily with more nuanced interpretations of provider accommodation of the underlying sociodemographic heterogeneity of Latinx patients and their diverse preferences. The onus lies on providers to assess an individual Latinx patient's English language skills, genetic health literacy, values, and preferences about provider involvement in decision‐making. We found evidence that Latinx patients reported difficulties communicating in clinical genetics settings, unmet need for provider discussion of cancer GT, concerns about unfair treatment, and that medical context and prior experiences with healthcare staff and institutions influenced GT decision‐making. Recent prenatal patient experience research also underscores positive experiences with GC in the U.S. contrasted with minimal prenatal care in Latin American countries, and the role of attentive care and social networks in patient decision‐making (Garza et al., 2020; Trainor et al., 2020). Such research implies that a medical context focused on building rapport between staff and patients, with effective methods to bridge communication challenges and comprehensive discussion of GC/GT with Latinx patients, perhaps with supportive Spanish‐language interpreters or navigators, would facilitate risk‐appropriate uptake of GC/GT.

Studies often lacked clear definitions of the characteristics of the study populations. Given the heterogeneity of Latinx populations, and the frequent lack of description of how ethnicity, socioeconomic, or immigrant status was defined or ascertained, results were subject to variability depending on the population sampled. Like Hunt et al. (2004), over half of the articles in the review did not specify the national or cultural origins of the Latinx population sampled. People with Mexican origins constitute over 60% of the Latinx population, which may influence findings about faith, fatalism, or non‐directiveness (Browner et al., 2003; Chalela et al., 2012; Griffiths & Kuppermann, 2008; Hamilton et al., 2016; Hooper et al., 2013; Ramirez et al., 2006; S. Thompson et al., 2015; Withers et al., 2019). Research on HBOC GT experience of Latinx of different origins highlights different GT awareness and desires about information and framing of risk. For instance, Cuban Americans emphasized urgency, and Mexican Americans, who have the lowest cancer GT awareness, requested details of testing procedures and not to be singled out or shamed (Vadaparampil et al., 2006, 2010). Those with low acculturation or LEP might require more fundamental information about the testing process (Floyd et al., 2016). Providers should anticipate that Latinx patients will have diverse ancestry, linguistic and cultural preferences, and socioeconomic status.

### 9.1 Study limitations

This study provides a synthesis of research about Latinx patient experience with GC/GT. Nevertheless, limitations impact validity, comprehensiveness, and generalizability. No formal quality assessment was performed because of the heterogeneity of included studies and the extraction and analysis were largely performed by the lead author. The findings may have missed pertinent research on Latinx patients due to exclusion of literature that was not peer reviewed and non‐English articles. A search strategy that used terms related to Hispanic/Latino and GC/GT in the title or abstract would not identified all research literature on GT used in medical practice (missing terms like non‐invasive prenatal screening).

Additionally, questions remain about how generalizable information gained from qualitative studies using convenience samples of patients, at‐risk family members, community members, or quantitative surveys of the public are to Latinx patients with various conditions. Research on both direct patient experience and more hypothetical scenarios were included in the review, despite the pitfalls of hypothetical research. This choice was made in an effort to capture a large subset of studies and the views of people who might not have access to healthcare, to glean findings on the experiences and potential impact of genetics in diverse clinical settings on Latinx patient populations. Variability in study findings may reflect the heterogeneity of people who self‐identify as Latinx and diverse sampling strategies, such as targeting safety‐net healthcare settings, underscoring the need for greater specificity when defining characteristics of the Latinx population sampled and comparative groups (Flanagin et al., 2021). A handful of studies may influence summarized findings, failing to capture the diversity of Latinx patients and their experiences. Because included studies were largely focused on Latinas' barriers to, attitudes about, or experiences with cancer risk assessment and prenatal testing, these findings may not apply to other types of GT, particularly clinical areas affecting men or children.

### 9.2 Avenues for future research

The predominance of research in urban prenatal or cancer care settings demonstrates avenues for future research to better understand how precision medicine approaches affect Latinx patients. Emphasis on cultural beliefs or low awareness of GT obscures the fact that poorer Latinx health outcomes, where they exist, likely originate from social determinants (Velasco‐Mondragon et al., 2016). As where people live, work, and their place in the social hierarchies is impactful on health outcomes, research might examine how social determinants of health interface with genetic susceptibilities, patient perspectives, and healthcare access or discrimination to more specifically elucidate Latinx health disparities.

Interpretation and other communication hurdles that impact GC/GT referrals suggest researching and training primary care providers or interpreters who serve Latinx patients to improve communication (Joseph et al., 2019; Kamara et al., 2018; Molina et al., 2019). The primary reasons for lower GT discussion and referrals with Latinx patients are unclear. Thus, future research might not only study communication but also elucidate how healthcare access, insurance coverage, medical context, comprehensiveness of family disease histories, and provider assumptions about genetic risk based on perceived ethnicity affect referrals.

Several articles stated categorically that non‐white participants expressed more concerns about privacy than NHW (American Society of Human Genetics, 2020; Clayton et al., 2018), but our research highlights that Latinx patients sometimes report less expectation of privacy than NHW (indicating others had a right to know GT results) (Chavez‐Yenter et al., 2021; Singer et al., 2004; Suther & Kiros, 2009). Based on our review, researchers might anticipate different findings about Latinx genetic privacy concerns depending on whether questions interrogate perceived obligation to divulge family history or GT results to physicians or family members (Carmichael et al., 2017; Kinney et al., 2010; Singer et al., 2004; Suther & Kiros, 2009; Tucker et al., 2006), or fears of discriminatory treatment (Glenn et al., 2012; Hall et al., 2005; Hamilton et al., 2016; Sussner et al., 2013). Many Latinx patients see GT collectivistically, as an empowering tool for families (Chavez‐Yenter et al., 2021), and they may be more prone than NHW patients to support providers sharing genetic information directly with family members (Kinney et al., 2010; MacDonald et al., 2008). Research should disambiguate aspects of genetic privacy, differentiating concerns about discrimination in the health sector from opinions about sharing of genetic information with providers and family members. A better understanding of Latinx patient sharing of genetics results could elucidate best practices for cascade testing.

## 10 CONCLUSION

U.S. healthcare often provides technologically intensive care to some, but rarely resources for implementation to all residents or reimbursement for time‐consuming medical conversations. To realize the ambition of precision medicine to improve healthcare outcomes without exacerbating health disparities requires improving the infrastructure to explain genetic risks as well as solicit and respond to diverse patient needs and contexts. In the spirit of providing tailored medical interventions equitably, this review suggests closer attention to provider approach and communication challenges for Latinx patients, especially those with LEP. Researchers and clinicians should be mindful of the inherent heterogeneity of the Latinx population, improving genetic services for the linguistically and socioeconomically marginalized. Attention to intergroup comparisons and socioeconomic factors such as income, unstable housing or work, education or health literacy, lack of insurance, and discrimination in healthcare settings would augment health disparities research seeking to improve genetically informed care for Latinx patients.

## AUTHOR CONTRIBUTIONS

**Heather A. Dron:** Data curation; formal analysis; investigation; project administration; software; supervision; validation; visualization; writing – original draft; writing – review and editing. **Daiana Bucio:** Formal analysis; writing – review and editing. **Jennifer L. Young:** Conceptualization; methodology; writing – review and editing. **Holly K. Tabor:** Conceptualization; formal analysis; writing – review and editing. **Mildred K. Cho:** Conceptualization; funding acquisition; methodology; supervision; writing – review and editing.

## Conflict of interest

Heather A. Dron, Jenni L. Young, Holly K. Tabor, and Mildred K. Cho report no conflicts of interest. Subsequent to her contributions to this article, Daiana Bucio was employed by and a shareholder of Invitae, Inc.

## Human studies and informed consent

No human research was carried out by the authors for this article.

## Animal studies

No non‐human animal studies were carried out by the authors for this article.

## Supporting information


**Appendix A:** Supplementary Information

**Appendix B:** Supplementary Information

## Data Availability

In addition to appendices summarizing findings, additional summary documents are available upon request.
