# Supplementary material for: Latinx attitudes, barriers, and experiences with genetic counseling and testing: A systematic review
Source: J Genet Couns. 2022 Oct 27;32(1):166–81. doi: 10.1002/jgc4.1632 (PMC10091969; doi:10.1002/jgc4.1632)
Supplement: Supplementary file 2 — Appendix B: Supplementary Information [file JGC4-32-166-s002.docx]

**Latinx & Genetic Counseling and Testing Search Queries By Database**

| **Name of database** | **number** | **date performed** |
| --- | --- | --- |
| Pubmed | 937 | 6/24/19 |
| Embase | 1,652 | 7/3/19 |
| psychinfo | 188 | 7/3/19 |
| CINAHL | 174 | 7/3/19 |
| Social Services Abstracts | 8 | 7/3/19 |
| **SUM** | 2,959 | - |
| **Unique articles** | 2,097 |  |

**PUBMED**

| Concept | Search | Result |
| --- | --- | --- |
| Genetic Testing | (((((((((genetic screening[Title/Abstract]) OR genetic testing[Title/Abstract]) OR genetic predictive testing[Title/Abstract]) OR genetic predisposition testing) OR genetic testings) OR Genetic testing[MeSH Terms]) OR genetic counseling[Title/Abstract]) OR prenatal genetic counseling[Title/Abstract]) OR preventative genetics[Title/Abstract]) OR genetic counseling[MeSH Terms] | 72,620 |
| Hispanic + Hispanic Americans | (((((((((((((((((((((((((((((((((((((((((((((((((((((((((hispanic[Title/Abstract]) OR latino[Title/Abstract]) OR Mexican[Title/Abstract]) OR Puerto Rican[Title/Abstract]) OR Cuban[Title/Abstract]) OR Domincan[Title/Abstract]) OR "Costa Rican"[Title/Abstract]) OR Guatemalan[Title/Abstract]) OR Honduran[Title/Abstract]) OR Nicaraguan[Title/Abstract]) OR Panamanian[Title/Abstract]) OR Salvadoran[Title/Abstract]) OR Argentinian[Title/Abstract]) OR Bolivian[Title/Abstract]) OR Chilean[Title/Abstract]) OR Columbian[Title/Abstract]) OR Ecuadorian[Title/Abstract]) OR Paraguayan[Title/Abstract]) OR Uruguayan[Title/Abstract]) OR Venezuelan[Title/Abstract]) OR Brazilian[Title/Abstract]) OR hispanics[Title/Abstract]) OR latinos[Title/Abstract]) OR latina[Title/Abstract]) OR latinas[Title/Abstract]) OR Hispanic American[Title/Abstract]) OR Hispanic Americans[Title/Abstract]) OR Latin American[Title/Abstract]) OR Latin Americans[Title/Abstract]) OR Central American[Title/Abstract]) OR Central Americans[Title/Abstract]) OR South American[Title/Abstract]) OR South Americans[Title/Abstract]) OR Caribbean[Title/Abstract]) OR Mexican American*[Title/Abstract]) OR Puerto Rican American*[Title/Abstract]) OR Cuban American*[Title/Abstract]) OR Dominican American*[Title/Abstract]) OR "Costa Rican American"[Title/Abstract]) OR Guatemalan American*[Title/Abstract]) OR Honduran American*[Title/Abstract]) OR Nicaraguan American*[Title/Abstract]) OR Panamanian American*[Title/Abstract]) OR Salvadoran American*[Title/Abstract]) OR Argentinean American*[Title/Abstract]) OR Bolivian American*[Title/Abstract]) OR Chilean American*[Title/Abstract]) OR Columbian American*[Title/Abstract]) OR Ecuadorian American*[Title/Abstract]) OR Paraguayan American*[Title/Abstract]) OR Uruguayan American*[Title/Abstract]) OR Peruvian American*[Title/Abstract]) OR Uruguayan American*[Title/Abstract]) OR Venezuelan American*[Title/Abstract]) OR Brazilian American*[Title/Abstract]) OR "Hispanic Americans"[MeSH Terms]) OR "Indians, Central America"[MeSH Terms]) OR "Indians, South America"[MeSH Terms] | 164,922 |
| Hispanic/Americans AND Genetic Testing | (((((((((((genetic screening[Title/Abstract]) OR genetic testing[Title/Abstract]) OR genetic predictive testing[Title/Abstract]) OR genetic predisposition testing[Title/Abstract]) OR genetic testings[Title/Abstract]) OR "Genetic testing"[MeSH Terms]) OR genetic counseling[Title/Abstract]) OR prenatal genetic counseling[Title/Abstract]) OR preventative genetics[Title/Abstract]) OR "genetic counseling"[MeSH Terms])) AND ((((((((((((((((((((((((((((((((((((((((((((((((((((((((((hispanic[Title/Abstract]) OR latino[Title/Abstract]) OR Mexican[Title/Abstract]) OR Puerto Rican[Title/Abstract]) OR Cuban[Title/Abstract]) OR Domincan[Title/Abstract]) OR "Costa Rican"[Title/Abstract]) OR Guatemalan[Title/Abstract]) OR Honduran[Title/Abstract]) OR Nicaraguan[Title/Abstract]) OR Panamanian[Title/Abstract]) OR Salvadoran[Title/Abstract]) OR Argentinian[Title/Abstract]) OR Bolivian[Title/Abstract]) OR Chilean[Title/Abstract]) OR Columbian[Title/Abstract]) OR Ecuadorian[Title/Abstract]) OR Paraguayan[Title/Abstract]) OR Uruguayan[Title/Abstract]) OR Venezuelan[Title/Abstract]) OR Brazilian[Title/Abstract]) OR hispanics[Title/Abstract]) OR latinos[Title/Abstract]) OR latina[Title/Abstract]) OR latinas[Title/Abstract]) OR Hispanic American[Title/Abstract]) OR Hispanic Americans[Title/Abstract]) OR Latin American[Title/Abstract]) OR Latin Americans[Title/Abstract]) OR Central American[Title/Abstract]) OR Central Americans[Title/Abstract]) OR South American[Title/Abstract]) OR South Americans[Title/Abstract]) OR Caribbean[Title/Abstract]) OR Mexican American*[Title/Abstract]) OR Puerto Rican American*[Title/Abstract]) OR Cuban American*[Title/Abstract]) OR Dominican American*[Title/Abstract]) OR "Costa Rican American"[Title/Abstract]) OR Guatemalan American*[Title/Abstract]) OR Honduran American*[Title/Abstract]) OR Nicaraguan American*[Title/Abstract]) OR Panamanian American*[Title/Abstract]) OR Salvadoran American*[Title/Abstract]) OR Argentinean American*[Title/Abstract]) OR Bolivian American*[Title/Abstract]) OR Chilean American*[Title/Abstract]) OR Columbian American*[Title/Abstract]) OR Ecuadorian American*[Title/Abstract]) OR Paraguayan American*[Title/Abstract]) OR Uruguayan American*[Title/Abstract]) OR Peruvian American*[Title/Abstract]) OR Uruguayan American*[Title/Abstract]) OR Venezuelan American*[Title/Abstract]) OR Brazilian American*[Title/Abstract]) OR "Hispanic Americans"[MeSH Terms]) OR "Indians, Central America"[MeSH Terms]) OR "Indians, South America"[MeSH Terms]) | 937 |

**EMBASE**

| Concept | Search | Result |
| --- | --- | --- |
| Genetic Testing | 'genetic screening':ti,ab,kw OR 'genetic testing':ti,ab,kw OR 'genetic predictive testing':ti,ab,kw OR 'genetic predisposition testing':ti,ab,kw OR 'genetic screenings':ti,ab,kw OR 'genetic screening'/exp OR 'genetic screening' OR 'genetic counseling':ti,ab,kw OR 'preventive genetics':ti,ab,kw OR 'prenatal genetic counseling':ti,ab,kw OR 'genetic counseling'/exp OR 'genetic counseling' | 116,563 |
| Hispanic + Hispanic Americans | 'hispanic'/exp OR 'hispanic' OR 'hispanic':ti,ab,kw OR 'hispanics':ti,ab,kw OR 'latino':ti,ab,kw OR 'latinos':ti,ab,kw OR 'latina':ti,ab,kw OR 'latinas':ti,ab,kw OR 'mexican':ti,ab,kw OR 'puerto rican':ti,ab,kw OR 'cuban':ti,ab,kw OR 'dominican':ti,ab,kw OR 'costa rican':ti,ab,kw OR 'guatemalan':ti,ab,kw OR 'honduran':ti,ab,kw OR 'nicaraguan':ti,ab,kw OR 'panamanian':ti,ab,kw OR 'salvadoran':ti,ab,kw OR 'argentinian':ti,ab,kw OR 'bolivian':ti,ab,kw OR 'chilean':ti,ab,kw OR 'columbian':ti,ab,kw OR 'ecuadorean':ti,ab,kw OR 'paraguayan':ti,ab,kw OR 'uruguayan':ti,ab,kw OR 'venezuelan':ti,ab,kw OR 'brazilian':ti,ab,kw OR 'south american':ti,ab,kw OR 'south americans':ti,ab,kw OR 'central american':ti,ab,kw OR 'central americans':ti,ab,kw OR 'hispanic american':ti,ab,kw OR 'hispanic americans':ti,ab,kw OR 'latin american':ti,ab,kw OR 'latin americans':ti,ab,kw OR 'caribbean':ti,ab,kw OR 'mexican american*':ti,ab,kw OR 'puerto rican american*':ti,ab,kw OR 'cuban american*':ti,ab,kw OR 'dominican american*':ti,ab,kw OR 'costa rican american*':ti,ab,kw OR 'guatemalan american*':ti,ab,kw OR 'honduran american*':ti,ab,kw OR 'nicaraguan american*':ti,ab,kw OR 'panamanian american*':ti,ab,kw OR 'salvadoran american*':ti,ab,kw OR 'argentinian american*':ti,ab,kw OR 'bolivian american*':ti,ab,kw OR 'chilean american*':ti,ab,kw OR 'columbian american*':ti,ab,kw OR 'ecuadorean american*':ti,ab,kw OR 'paraguayan american*':ti,ab,kw OR 'uruguayan american*':ti,ab,kw OR 'venezuelan american*':ti,ab,kw OR 'brazilian american*':ti,ab,kw | 216,910 |
| Hispanic/Americans AND Genetic Testing | ('genetic screening':ti,ab,kw OR 'genetic testing':ti,ab,kw OR 'genetic predictive testing':ti,ab,kw OR 'genetic predisposition testing':ti,ab,kw OR 'genetic screenings':ti,ab,kw OR 'genetic screening'/exp OR 'genetic screening' OR 'genetic counseling':ti,ab,kw OR 'preventive genetics':ti,ab,kw OR 'prenatal genetic counseling':ti,ab,kw OR 'genetic counseling'/exp OR 'genetic counseling') AND ('hispanic'/exp OR 'hispanic' OR 'hispanic':ti,ab,kw OR 'hispanics':ti,ab,kw OR 'latino':ti,ab,kw OR 'latinos':ti,ab,kw OR 'latina':ti,ab,kw OR 'latinas':ti,ab,kw OR 'mexican':ti,ab,kw OR 'puerto rican':ti,ab,kw OR 'cuban':ti,ab,kw OR 'dominican':ti,ab,kw OR 'costa rican':ti,ab,kw OR 'guatemalan':ti,ab,kw OR 'honduran':ti,ab,kw OR 'nicaraguan':ti,ab,kw OR 'panamanian':ti,ab,kw OR 'salvadoran':ti,ab,kw OR 'argentinian':ti,ab,kw OR 'bolivian':ti,ab,kw OR 'chilean':ti,ab,kw OR 'columbian':ti,ab,kw OR 'ecuadorean':ti,ab,kw OR 'paraguayan':ti,ab,kw OR 'uruguayan':ti,ab,kw OR 'venezuelan':ti,ab,kw OR 'brazilian':ti,ab,kw OR 'south american':ti,ab,kw OR 'south americans':ti,ab,kw OR 'central american':ti,ab,kw OR 'central americans':ti,ab,kw OR 'hispanic american':ti,ab,kw OR 'hispanic americans':ti,ab,kw OR 'latin american':ti,ab,kw OR 'latin americans':ti,ab,kw OR 'caribbean':ti,ab,kw OR 'mexican american*':ti,ab,kw OR 'puerto rican american*':ti,ab,kw OR 'cuban american*':ti,ab,kw OR 'dominican american*':ti,ab,kw OR 'costa rican american*':ti,ab,kw OR 'guatemalan american*':ti,ab,kw OR 'honduran american*':ti,ab,kw OR 'nicaraguan american*':ti,ab,kw OR 'panamanian american*':ti,ab,kw OR 'salvadoran american*':ti,ab,kw OR 'argentinian american*':ti,ab,kw OR 'bolivian american*':ti,ab,kw OR 'chilean american*':ti,ab,kw OR 'columbian american*':ti,ab,kw OR 'ecuadorean american*':ti,ab,kw OR 'paraguayan american*':ti,ab,kw OR 'uruguayan american*':ti,ab,kw OR 'venezuelan american*':ti,ab,kw OR 'brazilian american*':ti,ab,kw) | 1,650 |

**PsychInfo**

| Concept | Search | Result |
| --- | --- | --- |
| Genetic Testing | mainsubject.Exact.explode("Genetic Testing") OR TI(genetic screening OR genetic screenings OR genetic testing OR genetic predictive testing OR genetic predisposition testing OR genetic counseling OR preventive genetics OR prenatal genetic counseling) OR AB(genetic screening OR genetic screenings OR genetic testing OR genetic predictive testing OR genetic predisposition testing OR genetic counseling OR preventive genetics OR prenatal genetic counseling) | 9,142 |
| Hispanic + Hispanic Americans | mainsubject.Exact("hispanic americans") OR TI(hispanic OR hispanics OR latino OR Latinos OR latina OR latinas OR mexican OR puerto rican OR cuban OR dominican OR costa rican OR guatemalan OR honduran OR nicaraguan OR Panamanian OR salvadoran OR argentinian OR bolivian OR chilean OR columbian OR ecuadorean OR paraguayan OR uruguayan OR venezuelan OR brazilian OR south american OR south americans OR central american OR central americans OR hispanic american OR hispanic americans OR latin american OR Latin americans OR mexican american* OR puerto rican american* OR cuban american* OR dominican american* OR costa rican american* OR guatemalan american* OR honduran american* OR nicaraguan american* OR Panamanian american* OR salvadoran american* OR argentinian american* OR bolivian american* OR chilean american* OR columbian american* OR ecuadorean american* OR paraguayan american* OR uruguayan american* OR venezuelan american* OR brazilian american*) OR AB(hispanic OR hispanics OR latino OR Latinos OR latina OR latinas OR mexican OR puerto rican OR cuban OR dominican OR costa rican OR guatemalan OR honduran OR nicaraguan OR Panamanian OR salvadoran OR argentinian OR bolivian OR chilean OR columbian OR ecuadorean OR paraguayan OR uruguayan OR venezuelan OR brazilian OR south american OR south americans OR central american OR central americans OR hispanic american OR hispanic americans OR latin american OR Latin americans OR mexican american* OR puerto rican american* OR cuban american* OR dominican american* OR costa rican american* OR guatemalan american* OR honduran american* OR nicaraguan american* OR Panamanian american* OR salvadoran american* OR argentinian american* OR bolivian american* OR chilean american* OR columbian american* OR ecuadorean american* OR paraguayan american* OR uruguayan american* OR venezuelan american* OR brazilian american*) | 75,371 |
| Hispanic/Americans AND Genetic Testing | (mainsubject.Exact.explode("Genetic Testing") OR TI(genetic screening OR genetic screenings OR genetic testing OR genetic predictive testing OR genetic predisposition testing OR genetic counseling OR preventive genetics OR prenatal genetic counseling) OR AB(genetic screening OR genetic screenings OR genetic testing OR genetic predictive testing OR genetic predisposition testing OR genetic counseling OR preventive genetics OR prenatal genetic counseling)) AND (mainsubject.Exact("hispanic americans") OR TI(hispanic OR hispanics OR latino OR Latinos OR latina OR latinas OR mexican OR puerto rican OR cuban OR dominican OR costa rican OR guatemalan OR honduran OR nicaraguan OR Panamanian OR salvadoran OR argentinian OR bolivian OR chilean OR columbian OR ecuadorean OR paraguayan OR uruguayan OR venezuelan OR brazilian OR south american OR south americans OR central american OR central americans OR hispanic american OR hispanic americans OR latin american OR Latin americans OR mexican american* OR puerto rican american* OR cuban american* OR dominican american* OR costa rican american* OR guatemalan american* OR honduran american* OR nicaraguan american* OR Panamanian american* OR salvadoran american* OR argentinian american* OR bolivian american* OR chilean american* OR columbian american* OR ecuadorean american* OR paraguayan american* OR uruguayan american* OR venezuelan american* OR brazilian american*) OR AB(hispanic OR hispanics OR latino OR Latinos OR latina OR latinas OR mexican OR puerto rican OR cuban OR dominican OR costa rican OR guatemalan OR honduran OR nicaraguan OR Panamanian OR salvadoran OR argentinian OR bolivian OR chilean OR columbian OR ecuadorean OR paraguayan OR uruguayan OR venezuelan OR brazilian OR south american OR south americans OR central american OR central americans OR hispanic american OR hispanic americans OR latin american OR Latin americans OR mexican american* OR puerto rican american* OR cuban american* OR dominican american* OR costa rican american* OR guatemalan american* OR honduran american* OR nicaraguan american* OR Panamanian american* OR salvadoran american* OR argentinian american* OR bolivian american* OR chilean american* OR columbian american* OR ecuadorean american* OR paraguayan american* OR uruguayan american* OR venezuelan american* OR brazilian american*)) | 188 |

**Social Services Abstracts**

| Concept | Search | Result |
| --- | --- | --- |
| Genetic Testing | mainsubject.Exact.explode("Genetic Testing") OR TI(genetic screening OR genetic screenings OR genetic testing OR genetic predictive testing OR genetic predisposition testing OR genetic counseling OR preventive genetics OR prenatal genetic counseling) OR AB(genetic screening OR genetic screenings OR genetic testing OR genetic predictive testing OR genetic predisposition testing OR genetic counseling OR preventive genetics OR prenatal genetic counseling) | 435 |
| Hispanic + Hispanic Americans | mainsubject.Exact("hispanic americans") OR TI(hispanic OR hispanics OR latino OR Latinos OR latina OR latinas OR mexican OR puerto rican OR cuban OR dominican OR costa rican OR guatemalan OR honduran OR nicaraguan OR Panamanian OR salvadoran OR argentinian OR bolivian OR chilean OR columbian OR ecuadorean OR paraguayan OR uruguayan OR venezuelan OR brazilian OR south american OR south americans OR central american OR central americans OR hispanic american OR hispanic americans OR latin american OR Latin americans OR mexican american* OR puerto rican american* OR cuban american* OR dominican american* OR costa rican american* OR guatemalan american* OR honduran american* OR nicaraguan american* OR Panamanian american* OR salvadoran american* OR argentinian american* OR bolivian american* OR chilean american* OR columbian american* OR ecuadorean american* OR paraguayan american* OR uruguayan american* OR venezuelan american* OR brazilian american*) OR AB(hispanic OR hispanics OR latino OR Latinos OR latina OR latinas OR mexican OR puerto rican OR cuban OR dominican OR costa rican OR guatemalan OR honduran OR nicaraguan OR Panamanian OR salvadoran OR argentinian OR bolivian OR chilean OR columbian OR ecuadorean OR paraguayan OR uruguayan OR venezuelan OR brazilian OR south american OR south americans OR central american OR central americans OR hispanic american OR hispanic americans OR latin american OR Latin americans OR mexican american* OR puerto rican american* OR cuban american* OR dominican american* OR costa rican american* OR guatemalan american* OR honduran american* OR nicaraguan american* OR Panamanian american* OR salvadoran american* OR argentinian american* OR bolivian american* OR chilean american* OR columbian american* OR ecuadorean american* OR paraguayan american* OR uruguayan american* OR venezuelan american* OR brazilian american*) | 12,646 |
| Hispanic/Americans AND Genetic Testing | (mainsubject.Exact.explode("Genetic Testing") OR TI(genetic screening OR genetic screenings OR genetic testing OR genetic predictive testing OR genetic predisposition testing OR genetic counseling OR preventive genetics OR prenatal genetic counseling) OR AB(genetic screening OR genetic screenings OR genetic testing OR genetic predictive testing OR genetic predisposition testing OR genetic counseling OR preventive genetics OR prenatal genetic counseling)) AND (mainsubject.Exact("hispanic americans") OR TI(hispanic OR hispanics OR latino OR Latinos OR latina OR latinas OR mexican OR puerto rican OR cuban OR dominican OR costa rican OR guatemalan OR honduran OR nicaraguan OR Panamanian OR salvadoran OR argentinian OR bolivian OR chilean OR columbian OR ecuadorean OR paraguayan OR uruguayan OR venezuelan OR brazilian OR south american OR south americans OR central american OR central americans OR hispanic american OR hispanic americans OR latin american OR Latin americans OR mexican american* OR puerto rican american* OR cuban american* OR dominican american* OR costa rican american* OR guatemalan american* OR honduran american* OR nicaraguan american* OR Panamanian american* OR salvadoran american* OR argentinian american* OR bolivian american* OR chilean american* OR columbian american* OR ecuadorean american* OR paraguayan american* OR uruguayan american* OR venezuelan american* OR brazilian american*) OR AB(hispanic OR hispanics OR latino OR Latinos OR latina OR latinas OR mexican OR puerto rican OR cuban OR dominican OR costa rican OR guatemalan OR honduran OR nicaraguan OR Panamanian OR salvadoran OR argentinian OR bolivian OR chilean OR columbian OR ecuadorean OR paraguayan OR uruguayan OR venezuelan OR brazilian OR south american OR south americans OR central american OR central americans OR hispanic american OR hispanic americans OR latin american OR Latin americans OR mexican american* OR puerto rican american* OR cuban american* OR dominican american* OR costa rican american* OR guatemalan american* OR honduran american* OR nicaraguan american* OR Panamanian american* OR salvadoran american* OR argentinian american* OR bolivian american* OR chilean american* OR columbian american* OR ecuadorean american* OR paraguayan american* OR uruguayan american* OR venezuelan american* OR brazilian american*)) | 8 |

**CINAHL**

| Concept | Search | Result |
| --- | --- | --- |
| Genetic Testing | (MH "Genetic Counseling”) OR (MH "Genetic Screening”) OR TI (genetic counseling OR preventive genetics OR prenatal genetic counseling OR genetic testing OR genetic screening OR genetic screenings OR genetic predictive testing OR genetic predisposition testing) OR AB (genetic counseling OR preventive genetics OR prenatal genetic counseling OR genetic testing OR genetic screening OR genetic screenings OR genetic predictive testing OR genetic predisposition testing) | 17,249 |
| Hispanic + Hispanic Americans | TI (hispanic OR hispanics OR latino OR Latinos OR latina OR latinas OR mexican OR puerto rican OR cuban OR dominican OR costa rican OR guatemalan OR honduran OR nicaraguan OR Panamanian OR salvadoran OR argentinian OR bolivian OR chilean OR columbian OR ecuadorean OR paraguayan OR uruguayan OR venezuelan OR brazilian OR south american OR south americans OR central american OR central americans OR hispanic american OR hispanic americans OR latin american OR Latin americans OR mexican american* OR puerto rican american* OR cuban american* OR dominican american* OR costa rican american* OR guatemalan american* OR honduran american* OR nicaraguan american* OR Panamanian american* OR salvadoran american* OR argentinian american* OR bolivian american* OR chilean american* OR columbian american* OR ecuadorean american* OR paraguayan american* OR uruguayan american* OR venezuelan american* OR brazilian american*) OR AB (hispanic OR hispanics OR latino OR Latinos OR latina OR latinas OR mexican OR puerto rican OR cuban OR dominican OR costa rican OR guatemalan OR honduran OR nicaraguan OR Panamanian OR salvadoran OR argentinian OR bolivian OR chilean OR columbian OR ecuadorean OR paraguayan OR uruguayan OR venezuelan OR brazilian OR south american OR south americans OR central american OR central americans OR hispanic american OR hispanic americans OR latin american OR Latin americans OR mexican american* OR puerto rican american* OR cuban american* OR dominican american* OR costa rican american* OR guatemalan american* OR honduran american* OR nicaraguan american* OR Panamanian american* OR salvadoran american* OR argentinian american* OR bolivian american* OR chilean american* OR columbian american* OR ecuadorean american* OR paraguayan american* OR uruguayan american* OR venezuelan american* OR brazilian american*) | 48,598 |
| Hispanic/Americans AND Genetic Testing | ((MH "Genetic Counseling”) OR (MH "Genetic Screening”) OR TI (genetic counseling OR preventive genetics OR prenatal genetic counseling OR genetic testing OR genetic screening OR genetic screenings OR genetic predictive testing OR genetic predisposition testing) OR AB (genetic counseling OR preventive genetics OR prenatal genetic counseling OR genetic testing OR genetic screening OR genetic screenings OR genetic predictive testing OR genetic predisposition testing)) AND (TI (hispanic OR hispanics OR latino OR Latinos OR latina OR latinas OR mexican OR puerto rican OR cuban OR dominican OR costa rican OR guatemalan OR honduran OR nicaraguan OR Panamanian OR salvadoran OR argentinian OR bolivian OR chilean OR columbian OR ecuadorean OR paraguayan OR uruguayan OR venezuelan OR brazilian OR south american OR south americans OR central american OR central americans OR hispanic american OR hispanic americans OR latin american OR Latin americans OR mexican american* OR puerto rican american* OR cuban american* OR dominican american* OR costa rican american* OR guatemalan american* OR honduran american* OR nicaraguan american* OR Panamanian american* OR salvadoran american* OR argentinian american* OR bolivian american* OR chilean american* OR columbian american* OR ecuadorean american* OR paraguayan american* OR uruguayan american* OR venezuelan american* OR brazilian american*) OR AB (hispanic OR hispanics OR latino OR Latinos OR latina OR latinas OR mexican OR puerto rican OR cuban OR dominican OR costa rican OR guatemalan OR honduran OR nicaraguan OR Panamanian OR salvadoran OR argentinian OR bolivian OR chilean OR columbian OR ecuadorean OR paraguayan OR uruguayan OR venezuelan OR brazilian OR south american OR south americans OR central american OR central americans OR hispanic american OR hispanic americans OR latin american OR Latin americans OR mexican american* OR puerto rican american* OR cuban american* OR dominican american* OR costa rican american* OR guatemalan american* OR honduran american* OR nicaraguan american* OR Panamanian american* OR salvadoran american* OR argentinian american* OR bolivian american* OR chilean american* OR columbian american* OR ecuadorean american* OR paraguayan american* OR uruguayan american* OR venezuelan american* OR brazilian american*)) | 174 |
